# Effects of topical fluoride application on oral microbiota in young children with severe dental caries

**DOI:** 10.3389/fcimb.2023.1104343

**Published:** 2023-03-07

**Authors:** Zhengyan Yang, Ting Cai, Yueheng Li, Dan Jiang, Jun Luo, Zhi Zhou

**Affiliations:** ^1^ Department of Preventive Dentistry, Stomatological Hospital of Chongqing Medical University, Chongqing, China; ^2^ Chongqing Key Laboratory of Oral Biomedical Engineering of Higher Education, Department of Preventive Dentistry, Chongqing, China; ^3^ Chongqing Key Laboratory of Oral Diseases and Biomedical Sciences, Chongqing, China

**Keywords:** microbial community, fluoride, dental caries, high-throughput sequencing, saliva

## Abstract

While the effect of fluoride on severe early childhood caries (S-ECC) is clear, knowledge of how it influences the oral microbiota and the consequential effects on oral health is limited. In this cohort study, we investigated the changes introduced in the oral ecosystem before and after using fluoride varnish in 54- to 66-month-old individuals (n=90: 18 children were sampled at 5 different time points). 16S rDNA was amplified from bacterial samples using polymerase chain reaction, and high-throughput sequencing was performed using Illumina MiSeq platforms. Many pronounced microbial changes were related to the effects of fluoride varnishing. The health-associated *Bacteroides* and *Uncultured_bacterium_f_Enterobacteriaceae* were enriched in the saliva microbiome following treatment with fluoride varnishing. Co-occurrence network analysis of the dominant genera showed that different groups clearly showed different bacterial correlations. The PICRUSt algorithm was used to predict the function of the microbial communities from saliva samples. The results showed that starch and sucrose metabolism was greater after fluoride use. BugBase was used to determine phenotypes present in microbial community samples. The results showed that *Haemophilus* and *Neisseria* (phylum *Proteobacteria*) was greater before fluoride use. We conclude that the changes in oral microbiology play a role in fluoride prevention of S-ECC.

## Introduction

Dental caries is a chronic infectious disease resulting from many factors. Its causes are complex and diverse, but its formation is mainly due to the acid production of bacteria in the mouth, which leads to the dissolution and destruction of dental hard tissue (Vos et al., 2012). Currently, most experts think that dental caries is defined as a dysbiosis rather than a chronic infectious disease. ECC (Early childhood caries) is one of the most common chronic childhood diseases globally, affecting up to 73% of socioeconomically disadvantaged children (2018). The 4^th^ National Oral Health Surgery in mainland China in 2018 (Xiao et al., 2019) revealed that in the past decade, the prevalence of dental caries in young children in China was high, and the prevalence rate showed an upwards trend. The prevalence rate of dental caries in 5-year-old children was 71.9%, and the treatment of ECC, especially S-ECC, is complicated and difficult, with invasive and costly specialist treatment in the hospital under general anesthesia being the only option. Severe early childhood caries (S-ECC) occurred in children younger than three years old, those with one or more cavitated, missing, or filled smooth surfaces in primary maxillary anterior teeth from the ages of three to five, or those with a decayed, missing, or filled surface score ≥4 (age three), ≥5 (age four), or ≥6 (age five) (Losso et al., 2009). In this context, there is interest in simple treatments to halt the progress of cavities after tooth decay onset. Fluoride is recognized as effective in preventing caries. In many developed countries, the general decline in the incidence rate of caries has been largely attributed to the use of fluoride (Clark et al., 2020). From its physical and chemical mechanisms, the role of fluoride lies in the replacement reaction between fluoride ions and hydroxyapatite in the process of enamel mineralization. The formation of fluorapatite improves the hardness and acid resistance of enamel, which can reduce the formation of plaque (Takeuchi et al., 1996) and the incidence of caries.

Since Marsh (1994) proposed the ecological plaque hypothesis, further studies have found that due to changes in the oral environment, such as changes in sugar intake, diet or pH, the relatively balanced bacterial composition in biofilms can change significantly. The ecological balance of plaque is broken, leading to the occurrence of caries, and the occurrence and development of caries is the result of the imbalance of the microbial community. Research has also begun on the effects of fluoride on oral microbes. Studies have reported that long-term use of fluoride may cause changes in the flora (Yasuda et al., 2017). Fluoride can inhibit the growth of a variety of oral microorganisms, such as *Streptococcus sialis, Lactobacillus, Porphyromonas gingivalis, Streptococcus sanguis, Streptococcus mutans, and Candida albicans*, and different types of microorganisms have different sensitivities to fluoride (Kulik et al., 2015; Thomas et al., 2017; Maden et al., 2018). In addition, some studies have found that fluoride toothpaste can affect oral plaque biofilm. After treatment with fluoride toothpaste, the growth of *Streptococcus mutans* and *Porphyromonas gingivalis* was inhibited, while the number of *Streptococcus sanguis* increased (Cheng et al., 2017). Yasuda et al. (2017) established an animal model by simulating the fluoride-containing environment in which humans live. Through 16S rRNA gene amplification and genome sequencing, it was verified that fluoride interference has a selective effect on the composition of the oral microbial community in mice.

Previous studies have also found that fluorinated compounds inhibit bacterial growth by inhibiting the enzyme enolase, which catalyzes the conversion of 2-phosphoglycerate to phosphoenolpyruvate (the last step of anaerobic glycolysis), thereby improving oral health. Therefore, fluorinated compounds are crucial to microbial energy acquisition and growth (Marquis, 1995; Qin et al., 2006). However, how fluoride affects the whole oral microbiome and the changes in the oral microbiome after fluoride use have not been fully studied. This study mainly investigated how fluoride affects the microbial community in S-ECC saliva and its changes over time through Illumina MiSeq sequencing technology to better understand the microbial etiology of fluoride use.

## Materials and methods

### Subject selection

Patients with S-ECC were recruited from the same kindergarten in Yubei District of Chongqing, China. The inclusion criteria of subjects were as follows: (i) age from 54-66 months, (ii) no bad eating habits, (iii) no other bacterial infectious oral disease such as gingivitis or periodontitis, (iv) no antibiotic use within 2 months, (v) no partial denture and appliance, (vi) never having seen a dentist, and (vii) no systemic disease. All of the participants underwent a comprehensive oral examination, which included a professional assessment from a specialized dentist based on the standards of the World Health Organization “Oral health surveys: basic methods−5^th^ ed (World Health Organization, 2013).” In accordance with the timing of local fluoride varnish use (10 ml fluoride varnish containing 50 mg/ml NaF, Colgate-Palmolive UK For our study, participants received fluoride varnish treatment only at the first time. Before treatment, participants were instructed to brush their teeth, rinse their mouth, and remove any food debris. Dental surfaces were then dried and treated with fluoride using a small brush. Approximately 0.25 mL of fluoride was applied per participant, with emphasis on the grooves and adjacent surfaces.To maximize fluoride adherence, participants were advised to avoid drinking and rinsing for 30 minutes post-treatment. They were also told not to consume hard foods for four hours and to refrain from brushing their teeth that night. These measures ensured optimal fluoride absorption and efficacy. All the participants needed to collect saliva sample at 5 time points: HFB (Prefluoride saliva samples for subjects with high caries), HF1(Saliva samples taken 1 day after fluoride application in subjects with high caries), HF3(Saliva samples taken 3 days after fluoride application in subjects with high caries), HF7 (Saliva samples taken 7 days after fluoride application in subjects with high caries), and HF14 (Saliva samples taken 14 days after fluoride application in subjects with high caries). During this observation period, all the participants were provided with the same toothbrush and toothpaste without fluoride, and they were also forbidden to use mouth rinse, dental floss and so on containing fluoride. These participants’ parents or grandparents were sufficiently informed about the aims of the research and provided written informed consent according to the recommendations of the Ethics Committee of the Stomatological Hospital of Chongqing Medical University (CQHS-REC-2018(LSNO.22)).

### Sample collection

All of the participants were required to avoid eating, drinking, and brushing their teeth 2 hours before taking samples and then rinsed their mouths with sterile water. Unstimulated saliva was collected, transferred to sterile 1.5 mL microcentrifuge tubes, and frozen at -80°C until further processing.

### DNA extraction, PCR amplification and Illumina MiSeq sequencing

Based on the manufacturer’s protocol, microbial DNA was extracted from all the specimens by the PowerSoil^®^ DNA Isolation Kit. Primers 338F (5′-ACTCCTACGGGAGGCAGCAG-3′) and 806R (5′-GGACTACHVGGGTWTCTAAT-3′) were used to amplify the V3-V4 hypervariable regions of the bacterial 16S rRNA gene using PCR on the Veriti 96-Well Thermal Cycler (GeneAmp 9902, ABI, USA). Target area PCR was conducted with the following program: initial denaturation at 98°C for 2 mins, 30 cycles of denaturation at 98°C for 30 s, annealing at 50°C for 30 s, and elongation at 72°C for 60 s, and a final extension at 72°C for 5 mins, followed by storage at 4°C. Target area PCR was performed in triplicate with a 30 µL mixture containing 15 µL KOD FX Neo Buffer, 6 µL dNTPs (2 mM each), 0.9 µL Vn F (10 µM)/Vn R (10 μM), 0.6 µL KOD FX Neo and 50 ng ± 20% template DNA. Finally, ddH2O was used to fill to a 30 µL volume. Solexa PCR was conducted with the following program: initial denaturation at 98°C for 30 s, 10 cycles of denaturation at 98°C for 10 s, annealing at 65°C for 30 s, and elongation at 72°C for 60 s, and a final extension at 72°C for 5 mins.

Solexa PCR was performed in triplicate with a 20 µL mixture containing 5 µL target area PCR products, 10 µL of 2×Q5 HF MM, and 2.5 µL MPPI-a (2 µM)/MPPI-b (2 μM). The final PCR products were extracted from a 1.8% agarose gel under a voltage of 120 V. After 40 mins, column purification was performed by using an OMEGA DNA purification column. The PCR products were purified, quantified, and homogenized to form a sequencing library. The built libraries were first subjected to library quality inspection, and the qualified libraries were sequenced by Illumina NovaSeq 6000 PE250.

### Processing of sequencing data

Primary FastQ files were divided into multiple files for processing and quality-filtered by Trimmomatic (Edgar Robert, 2013). The following standards were used for sequence combination: (i) Reads containing any site with an average quality score <20 over a 50 bp sliding window were removed. (ii) The primers must be perfectly matched, allowing the mismatch of two nucleotides, and reads with ambiguous bases were removed. (iii) Sequences with an overlap longer than 10 bp were merged based on their overlapping sequence (Jiang et al., 2019).

By USEARCH (Bolger et al., 2014) (version 10.0), operational taxonomic units (OTUs) were clustered with a 97% similarity cut-off. Additionally, chimeric sequences were recognized and deleted by UCHIME (version 8.1) (Edgar et al., 2011). By comparing the RDP Classifier algorithm (Wang et al., 2007) (version 2.2, http://sourceforge.net/projects/rdpclassifier/) against the Silva (Quast et al., 2012) 16S rRNA (Release128, http://www.arb-silva.de) database, the taxonomy of each 16S rRNA gene sequence was analyzed based on an 80% confidence threshold.

### Bioinformatics and statistical analysis

Bioinformatics analysis was conducted using QIIME. The alpha diversity indices of Shannon, Simpson, Chao, ACE and PD_whole_tree were calculated at 97% identity by Mothur (Schloss et al., 2009) version v.1.30. Beta diversity analysis was performed by principal coordinate analysis (PCoA) based on Bray-Curtis distances at the OTU level. A hierarchical clustering analysis based on weighted UniFrac distances was also conducted (Lozupone et al., 2007). Analysis of similarities (ANOSIM) was performed using the vegan package in the R language and drawn with Python; unweighted UniFrac distances were used to compare different groups. STAMP v2.1.3 with Welsh’s t test (p < 0.05) and Kruskal-Wallis were used to compare the relative abundance of predominant bacteria between different groups. A P value of <0.05 was considered to be statistically significant. A Venn diagram was used to define the core microbiome at the species level using Mothur (Schloss et al., 2009). Linear discriminant analysis of effect size (LEfSe) was conducted to define the biomarkers of the groups. The threshold on the logarithmic LDA score for distinguishing features was set to 3 (Segata et al., 2011). Receiver operating characteristic (ROC) curve analysis was performed on the Tutools platform (https://www.cloudtutu.com), a free online data analysis website. Co-occurrence analysis among the genera was performed with Python, and co-occurrence analysis of the 80 richest genera of each group was performed at the same time. The phylogenetic investigation of communities by reconstruction of unobserved states (PICRUSt2) program was used to predict 16S rRNA-based data from high-throughput sequencing and to further analyze the composition and differential Kyoto Encyclopedia of Genes and Genomes (KEGG) metabolic pathways in the context of IMG microbial genome data. STAMP v2.1.3 with Welsh’s t test (p < 0.05) was used to compare the pathways between different groups. BugBase normalized the OTU by the predicted 16S copy number and then predicted the microbial phenotype (Ward et al., 2017) using the provided precomputed files. Differences were considered significant when *P* < 0.05 and extremely significant when *P* < 0.01. SPSS 25.0 software (SPSS Inc., Chicago, IL, USA) was used for statistical analysis. The raw data will be made available by the authors without undue reservation to any qualified researcher.

## Results

### Sequence information

**Figure 1:**
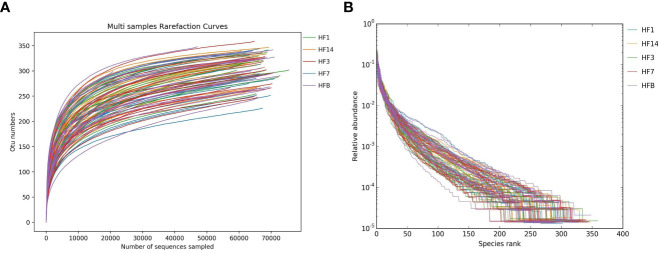
Alpha diversity analysis of saliva microorganisms. Rarefaction curve **(A)** and rank abundance curve **(B)** of saliva floral of samples.

A total of 18 preschool children were enrolled in this study at the end of the follow-up period (**Table 1**). The mean age at baseline for preschool children was 60.2 months. After Illumina MiSeq sequencing, 6,320,730 effective sequences were acquired from 90 saliva samples, with an average of 70,230 sequences per sample. The average length of the sequences was 423 bp. Ninety-seven percent qualified sequences were clustered, and 2,731 (**Appendix 1**) operational taxonomic units (OTUs) were obtained. Good’s coverage of the generated OTUs reached 99.9%. In the rarefaction curve, each curve first rose sharply and then flattened out with an increase in the number of sequences, indicating that the sequencing quantity was sufficient to cover all species in the samples (**Figure 1A**). There was a high evenness of saliva microbial composition in the samples, as seen in the rank abundance curve (**Figure 1B**).

**Table 1:** The (HFB) participants’ caries status (decayed teeth).

Samples	Age (months)	Sex	dmft	dmfs
AHDB	54	Female	8	17
AHPB	55	Female	13	48
BHDB	60	Female	8	27
BHPB	57	Male	18	56
CHDB	62	Male	16	51
CHPB	64	female	8	20
DHDB	63	female	8	18
DHPB	58	female	12	21
EHDB	59	male	12	20
EHPB	63	male	8	20
FHDB	66	male	10	27
FHPB	66	male	12	25
GHDB	57	male	9	29
GHPB	58	female	8	28
HHDB	58	female	8	25
HHPB	60	female	8	17
IHDB	61	female	10	31
IHPB	63	male	8	25

### Alpha and beta diversity analysis based on 16S rRNA sequencing

**Figure 2:**
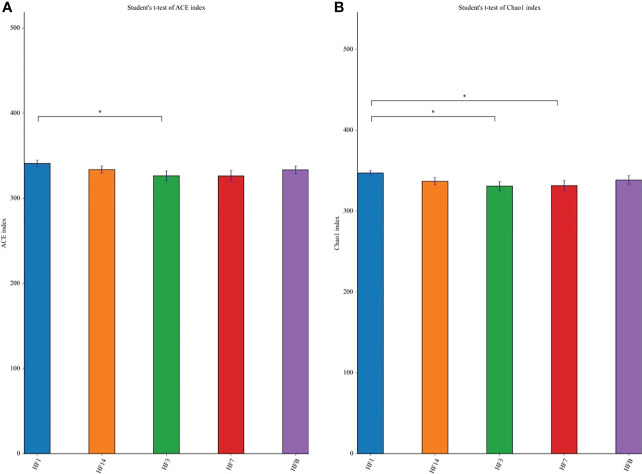
The significance of the difference in the Ace index **(A)** and Chao1 **(B)** index between each group. The significant differences were evaluated by t test (*P < 0.05, the asterisk indicates statistical significance).

**Figure 3:**
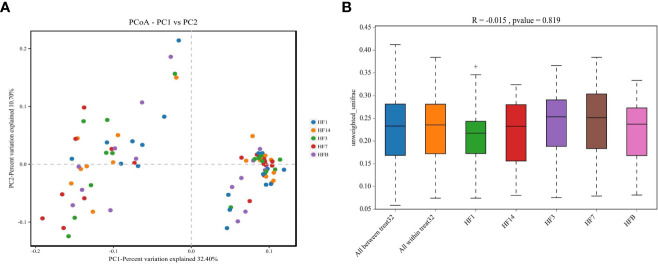
PCoA based on Bray-Curtis distances at the OTU level at 97% identity. Each sample is represented by a dot. Circles in different colors represent different groups. PC1 explained 32.4% of the variation observed, and PC2 explained 10.7% of the variation observed **(A)**. ANOSIM was based on unweighted UniFrac distances between the five groups (P=0.819) **(B)**.

The Shannon, Simpson, Chao, ACE and PD_ whole_tree alpha diversity was calculated to analyze the diversity and abundance of all samples. Student’s t test was performed to compare the saliva samples among the five groups (HFB, HF1, HF3, HF7, and HF14). **Table 2** and **Figure 2** show that the difference between HF1 and HF3 is statistically significant for ACE and Chao1, the difference between HF1 and HF7 is statistically significant for Chao1, and the other groups are not statistically significant for the other diversity indices. The Chao1 and Ace indices measure the species abundance, that is, the number of species. The results of this study showed that only the difference between HF1 and HF3 and HF7 was statistically significant but when compared with HFB, the difference has no statistically significant, indicating that the diversity and richness of the bacterial communities across the five groups were basically similar. Only in terms of species richness, with the use of local fluoride, did the species richness first increase and then decrease until it returned to a level close to that before the use of fluoride. PCoA and ANOSIM were used to evaluate the similarity in the microbial community structure among the five groups (**Figure 3**). PCoA and ANOSIM results showed that there was no significant difference in bacterial composition among the five groups. This finding suggested that children’s oral microbial community structure was similar before and after topical fluoride application.

**Table 2:** Alpha diversity indices of different groups.

Group	Shannon	Simpson	Chao1	ACE	PD_whole_tree
HFB	5.43 ± 0.12	0.95 ± 0.004	338.46 ± 5.53	333.53 ± 4.46	19.64 ± 0.34
HF1	5.41 ± 0.11	0.95 ± 0.004	347.22 ± 2.98	340.99 ± 3.89	19.92 ± 0.25
HF3	5.20 ± 0.98	0.94 ± 0.003	330.90 ± 5.60*	326.44 ± 5.79*	19.44 ± 0.38
HF7	5.27 ± 0.10	0.95 ± 0.004	331.57 ± 6.27*	326.43 ± 6.19	19.28 ± 0.42
HF14	5.32 ± 0.07	0.95 ± 0.003	336.89 ± 4.51	333.82 ± 4.16	19.79 ± 0.31

*Compared with the HF1 group, *P*<0.05.

### Bacterial community structure

**Figure 4:**
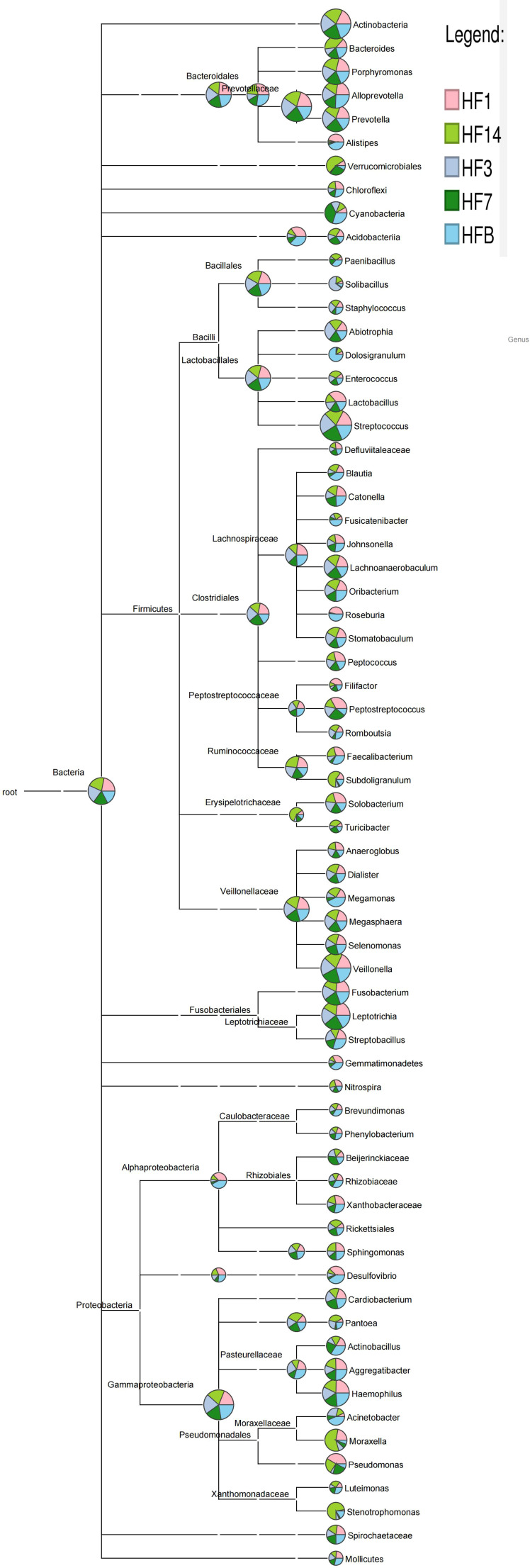
Phylogenetic tree of the 68 most abundant genera. Each branch represents a taxon, the length shows the phylogenetic distances between two taxa, and different colors represent different phyla. The bar plot on the right side shows the relative abundance of each genus in the five groups.

**Figure 5:**
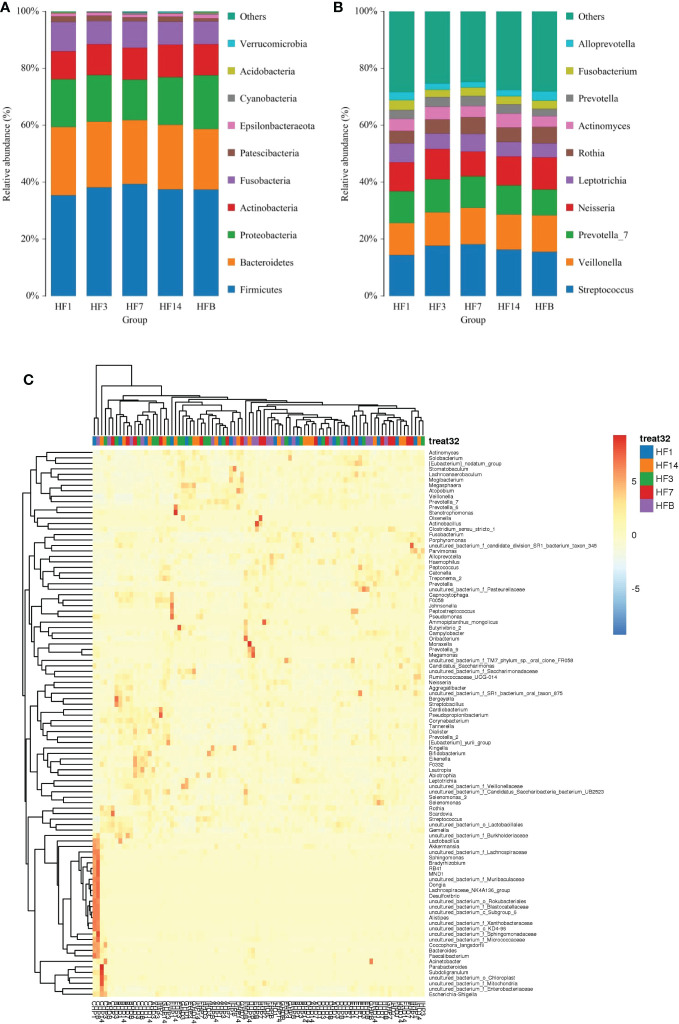
The distributions of the predominant bacteria. **(A)** Results at the phylum level. **(B)** Results at the genus level. The predominant taxa (relative abundance >2% on average) are shown. **(C)** Heatmap analysis. Each column represents a sample, and each row represents a genus. The cluster trees of genera and samples are shown on the left and upper sides, respectively. Different colors represent different relative abundances.

OTUs were distributed among 16 phyla, 26 classes, 56 orders, 93 families, 173 genera, and 218 species. The phylogenetic trees of the 68 most abundant genera were constructed, in which the taxonomic composition and abundance can be observed (**Figure 4**). The top abundant phyla were *Firmicutes* (37.6%), *Bacteroidetes* (22.7%), *Proteobacteria* (16.6%), *Actinobacteria* (10.9%), *Fusobacteria* (8.7%), Patescibacteria (1.7%), Epsilonbacteraeota (1.1%), Cyanobacteria (3.7%), Acidobacteria (1.5%) and Spirochaetes (1.5%), together comprising 98.2% of the total sequences (**Figure 5A**). The most abundant genera were *Streptococcus* (16.4%), *Veillonella* (12.2%), *Prevotella_7* (10.7%), *Neisseria* (10.2%), *Leptotrichia* (5.6%), Rothia (5.3%), *Actinomyces* (4.2%), *Prevotella* (3.2%), *Fusobacterium* (2.9%), and Alloprevotella (2.5%), accounting for 73.2% of the total (**Figure 5B**). The predominant bacteria were largely consistent among the five groups, but different relative abundances could be observed. **Figure 5C** represents a heatmap showing the relative abundances of these predominant genera in each sample, as well as the cluster trees of the genera and samples. The microbial compositions of each group were not obviously separated, consistent with the results of beta diversity and ANOSIM based on 16S rRNA sequencing.

**Figure 6:**
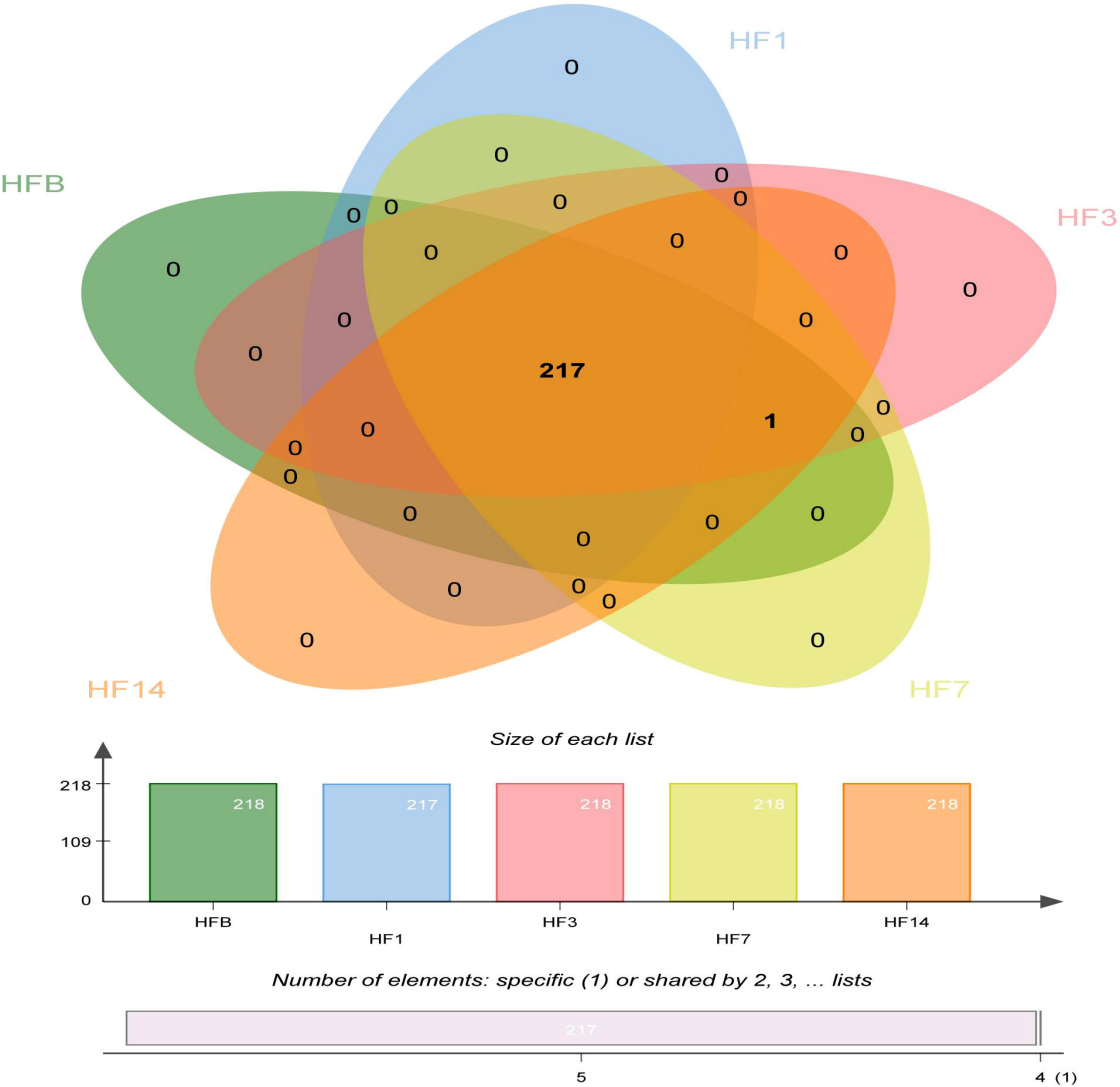
Venn diagram at the species level. Different colors represent different groups. The overlaps represent the common taxa between groups, and the nonoverlapping portions represent unique taxa in each group.

A Venn diagram was made to define the core microbiome, which was detected in most individuals at the species level. We identified 217 species in the HF1 group and 218 species in the other groups (**Figure 6**). Among them, 217 species were shared, occupying 99.5% of all the species detected, and 1 species (*uncultured_bacterium_g_Comamonas*) was unique to the HF1 group, indicating a stable composition of the microbiome in each group.

### Similarities and dissimilarities in bacterial compositions

**Figure 7:**
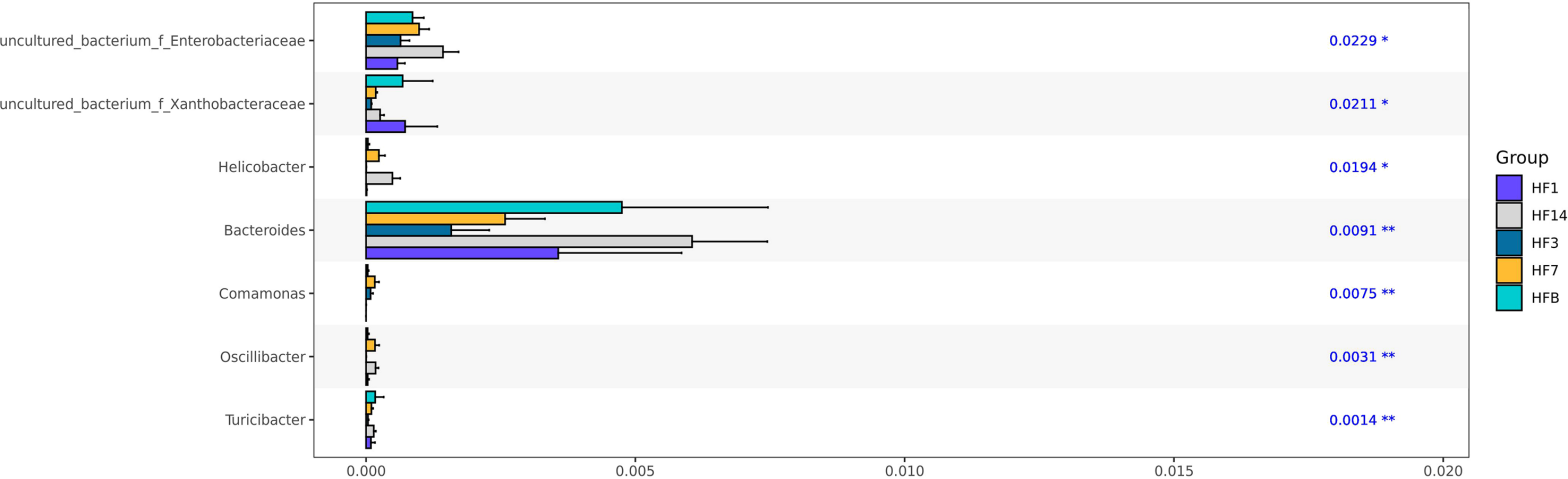
Kruskal-Wallis test bar plot at the genus level of five groups. (*P < 0.05, **P<0.01; the asterisk indicates statistical significance).

**Figure 8:**
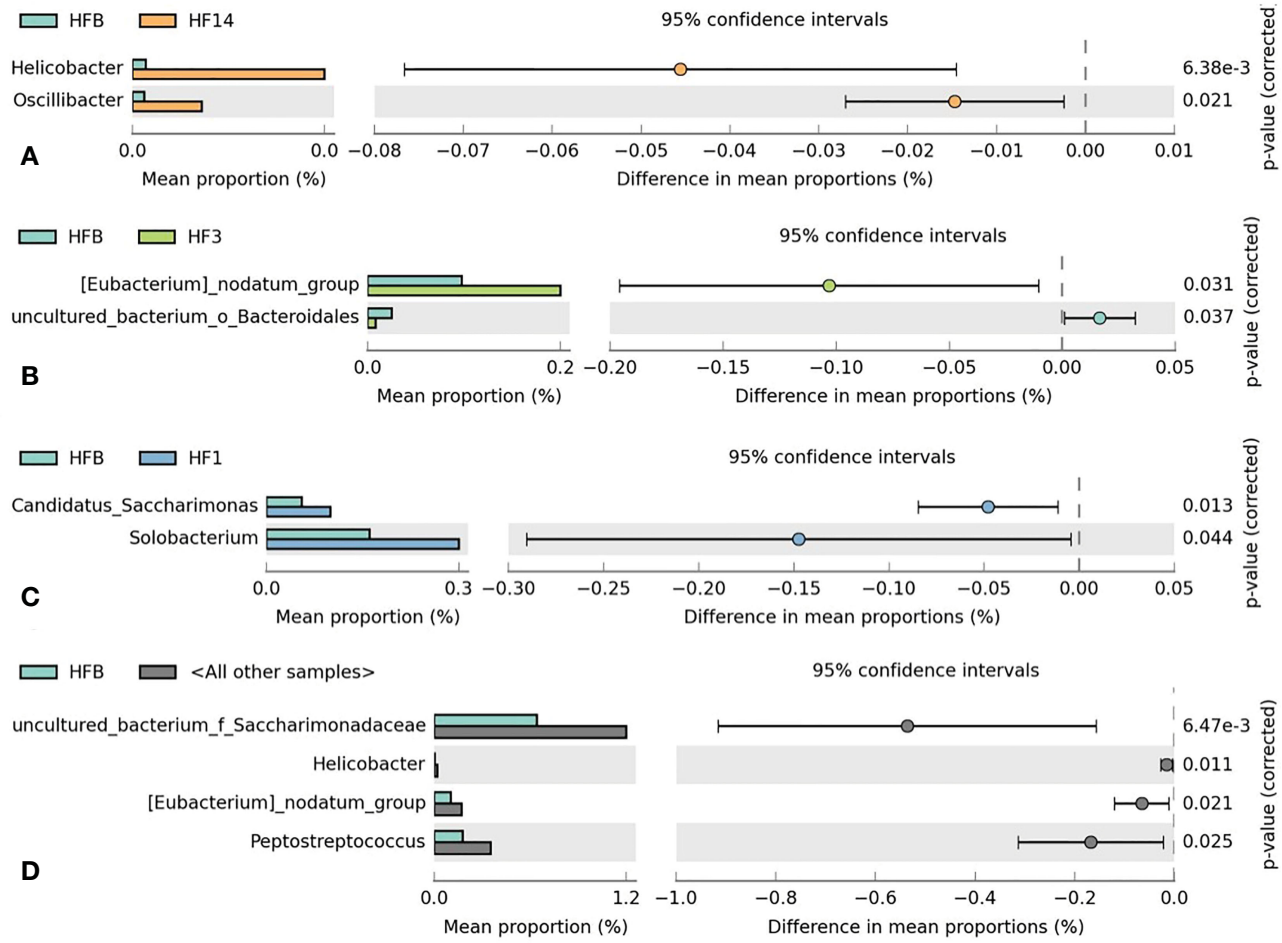
Welch’s t test bar plot at the genus level. **(A)** Results of the HFB and HF14 groups. **(B)** Results of the HFB and HF3 groups. **(C)** Results of the HFB and HF1 groups. **(D)** Results of the HFB and all other samples. Only the results that were significantly different (P < 0.05) are shown.

Kruskal-Wallis analysis (**Figure 7**) of the 5 groups of samples at the genus level showed that *Oscillibacter, Bacteroides, Helicobacter, and uncultured_ bacterium_ f_ Enterobacteriaceae* had the highest relative abundance in the HF14 group; *Turicibacter* had the highest relative abundance in the HFB group; and *Comamonas* had the highest relative abundance in the HF7 group. The relative abundance of *uncultured_ bacterium_ f_ Xanthobacteraceae* in the HF1 group was the highest, and the difference was statistically significant (*P*<0.05). STAMP difference analysis was performed between the HFB group and the other four groups of samples at the genus level. The results showed that compared with the HFB group, the relative abundance of *Helicobacter* and *Oscillibacter* in the HF14 group was higher. The relative abundance of *[Eubacterium]_nodatum_group* and *uncultured_bacterium_o_Bacteroidales* in the HF3 group was higher, and the relative abundance of *Candidatus_Saccharimonas* and *Solobacterium* in the HF1 group was higher. Compared with all groups after local fluoride use, the relative abundance of *Helicobacter, uncultured_bacterium_f_Saccharimonadaceae, Peptostreptococcus* and *[Eubacterium]_nodatum_group* was higher, and the above differences were statistically significant (*P* < 0.05) (**Figure 8**). No significant difference was found between the HFB group and HF7 group at the genus level.

**Figure 9:**
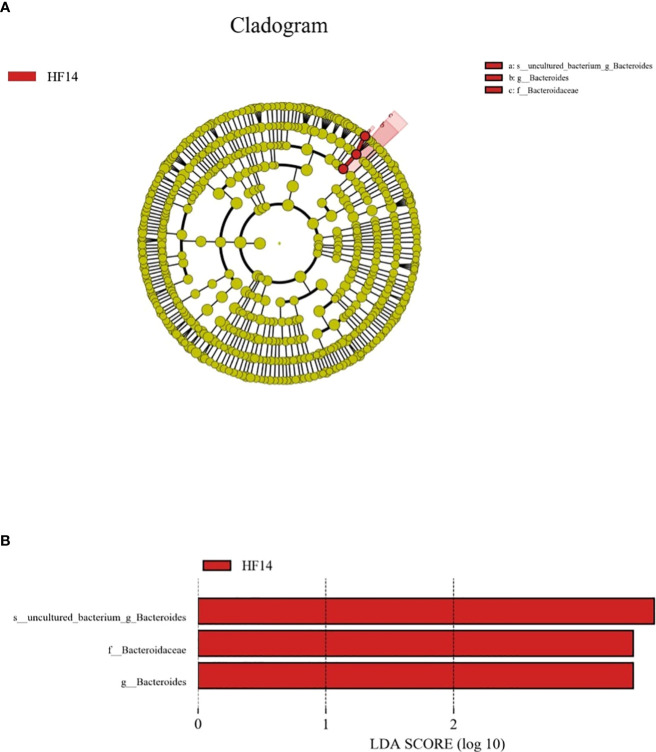
The potential biomarkers were defined by LEfSe. **(A)** Cladogram for taxonomic representation of significant differences between the five groups. The colored nodes from the inner to the outer circles represent taxa from the phylum to the genus level. The significantly different taxa are signified by different colors representing the five groups. **(B)** Histogram of the LDA scores for differentially abundant features among the groups. The threshold on the logarithmic LDA score for discriminative features was set to 3.0.

LEfSe was used to explain the characteristics of differences among groups HFB, HF1, HF3, HF7, and HF14. **Figure 9** shows a branching diagram of potential biomarkers representing different groups. At the genus level, *Bacteroides* was remarkably enriched in the HF14 group. At the species level, the relative abundance of *uncultured_bacterium_g_Bacteroides* was higher in the HF14 group (LEfSe LDA = 3, P < 0.05). This result indicated that the higher relative abundance of *Bacteroides* may be related to the local application of fluoride.

**Figure 10:**
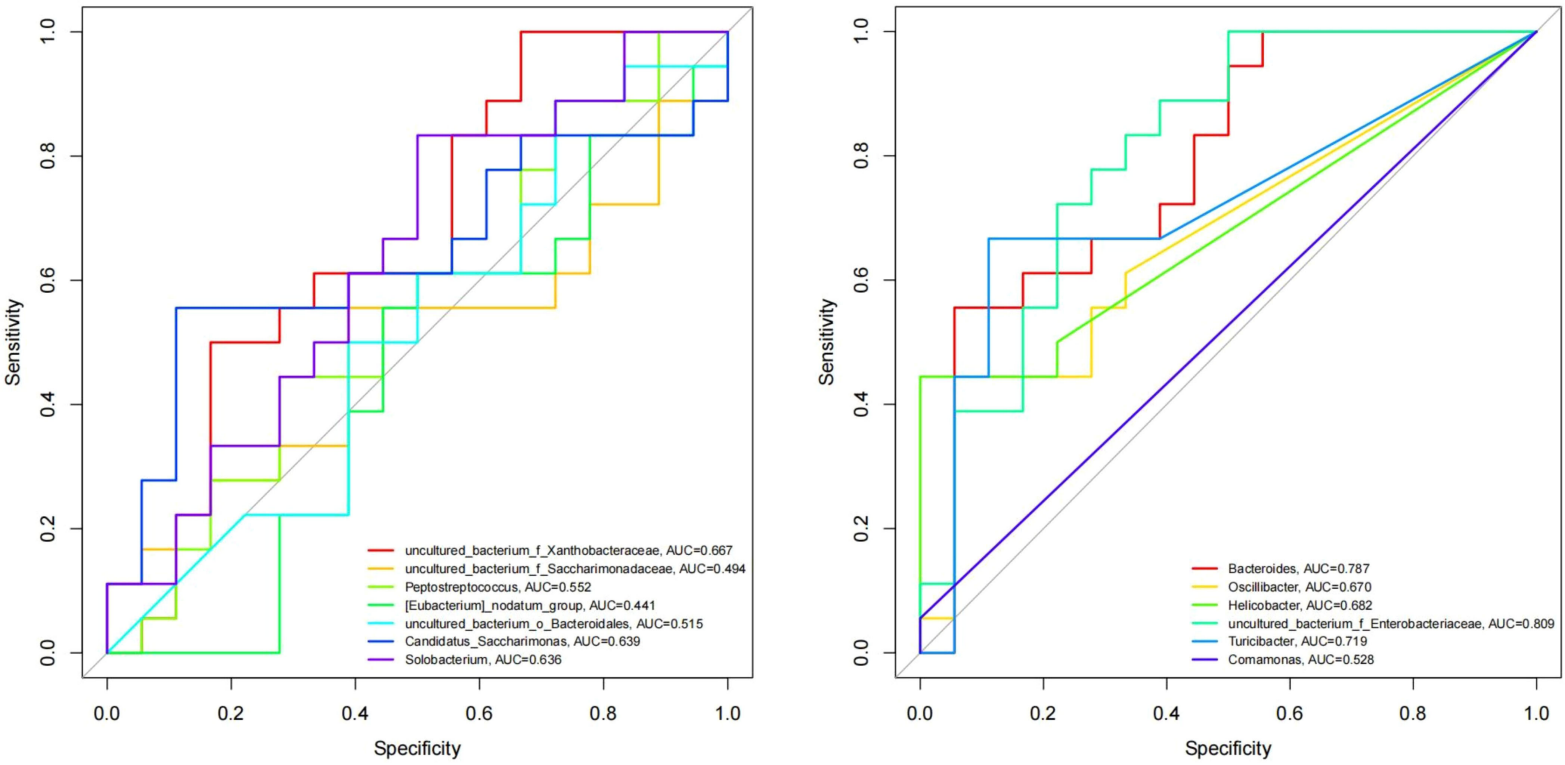
ROC curve model of differentially abundant genera. Different colored lines represent different genera.

The abscissa of the ROC curve model is the false-positive rate, and the ordinate is the true positive rate, which can simultaneously reflect the sensitivity, specificity and accuracy of the results. AUC is the area under the ROC curve, and the AUC value is usually between 0.5 and 1; it is generally believed that the AUC has a certain diagnostic value in the range of 0.7-0.9. At the genus level, ROC curve analysis was performed on the differential species screened by LEfSe, STAMP and Kruskal-Wallis, and the AUC was calculated to assess changes in microbial diversity after fluoride use. As shown in **Figure 10**, the differences between *Bacteroides* (AUC=0.787), *Turicibacter* (AUC=0.719) and *uncultured_bacterium_f_Enterobacteriaceae* (AUC=0.809) can be used as biomarkers to speculate the changes in microbial diversity after fluoride use.

### Network analysis and function prediction

**Figure 11:**
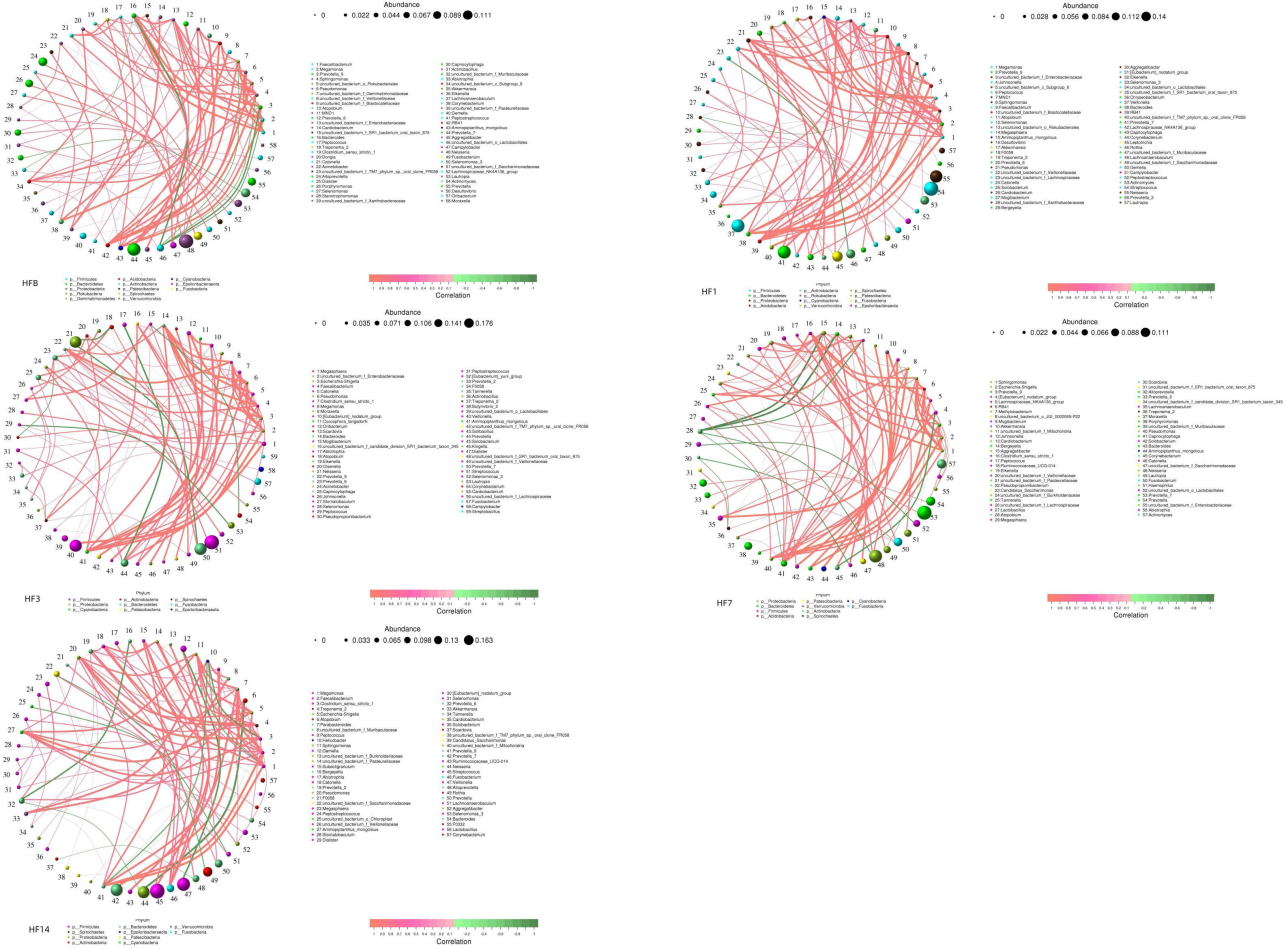
Network analysis showing the interactions between genera (|SpearmanCoef| > 0.1 and P < 0.05). Bacterial interactions of the five different groups (the 80 richest genera). The size of the node is proportional to the genera abundance. Node color corresponds to phylum taxonomic classification. Edge color represents positive (red) and negative (green) correlations.

Co-occurrence analysis was performed to recognize interactions among genera in different groups. The top 80 genera with high relative abundance were found to have complex interactions in each group (**Figure 11**). Different groups clearly showed different bacterial correlations, and there were 7, 3, 8, 13 and 13 negative correlations in the HFB, HF1, HF3, HF7, and HF14 groups, respectively. In the HFB group, *Bacteroides* had a positive correlation with *Faecalibacterium, Megamonas, Prevotella_9, uncultured_bacterium_f_Enterobacteriaceae, Pseudomonas* and *Ammopiptanthus_mongolicus* and a negative correlation with *uncultured_bacterium_o_Lactobacillales* and *Abiotrophia*. In the HF1 group, *Bacteroides* had a positive correlation with *Prevotella_9, Megamonas, Faecalibacterium, Sphingomonas, Pseudomonas* and *Ammopiptanthus_mongolicus*. In the HF3 group, *Bacteroides* had a positive correlation with *Escherichia-Shigella, Faecalibacterium, Pseudomonas, Megamonas, Ammopiptanthus_mongolicus, Prevotella_9, uncultured_bacterium_f_Enterobacteriaceae* and *Clostridium_sensu_stricto_1*. In the HF7 group, *Bacteroides* had a positive correlation with *Lachnospiraceae_NK4A136_group, Akkermansia and uncultured_bacterium_f_Muribaculaceae*. In the HF14 group, *Bacteroides* had a positive correlation with *Helicobacter and Akkermansia*. Compared to that in the HFB group, *Bacteroides* in the HF1, HF3, HF7 and HF14 groups only exhibited a positive correlation.

**Figure 12:**
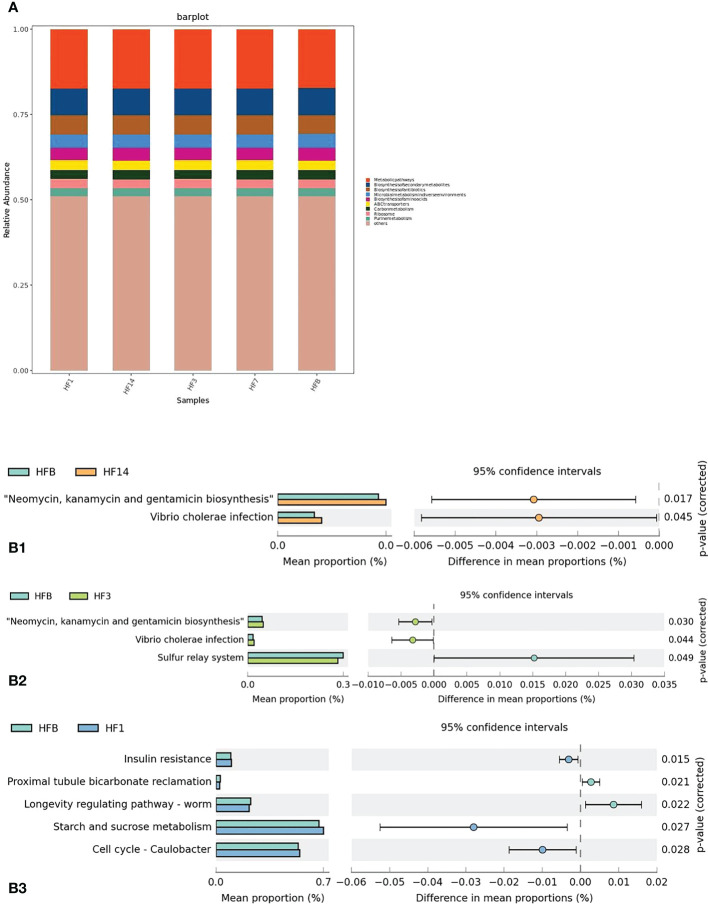
Function prediction by PICRUSt. **(A)** The compositions of KEGG functions in the five groups (in class 3). **(B)** Welch’s t test bar plot of the KEGG results. (B1) Results of the HFB and HF14 groups in Class 3. (B2) Results of the HFB and HF3 groups in Class 3. (B3) Results of the HFB and HF1 groups in Class 3. Only the results that were significantly different (P < 0.05) are shown.

PICRUSt2 was used to help understand the function of microbial communities in saliva samples. A bar graph (**Figure 12A**) showed that the saliva samples from the five groups had similar KEGG maps, suggesting that the function of microbiota in the five groups was similar. However, STAMP analysis showed that there was a difference in the function of microbiota between each group. Compared with HFB in class 3, there was a higher relative abundance of neomycin, kanamycin and gentamicin biosynthesis and *Vibrio cholerae* infection in the HF14 and HF3 groups; a higher relative abundance of sulfur metabolism in the HFB group; a higher relative abundance of insulin resistance, starch and sucrose metabolism, and cell cycle - Caulobacter in the HF1 group; and a higher relative abundance of proximal tubule bicarbonate reclamation and longevity regulating pathway in the HFB group (*P* < 0.05) (**Figure 12B**).

**Figure 13:**
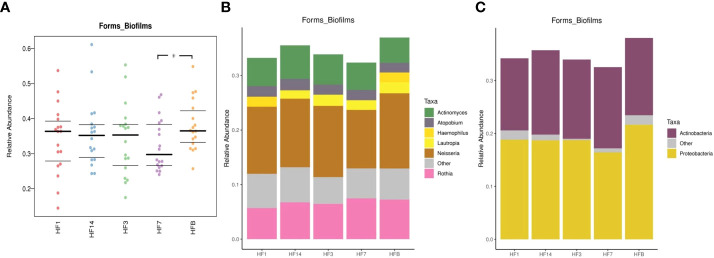
**(A)** BugBase was used to predict the proportion of forms_biofilm bacteria within the microbiomes of each group (n=18, * represents a significant difference, P=0.037<0.05); **(B)** the corresponding OTU contribution plots of the relative abundance of genera possessing each phenotype are also shown; **(C)** the corresponding OTU contribution plots of the relative abundance of phyla possessing each phenotype are also shown.

BugBase was used to determine phenotypes present in microbiota samples. The nine phenotypes included aerobic, anaerobic, contain_mobile_elements, facultative_anaerobic, forms_biofilms, gram_negative, gram_positive, potentially_pathogenic, and stress_tolerant. We performed pairwise comparisons of five samples using Mann-Whitney-Wilcoxon tests, and differences in the proportion of forms_biofilms bacteria were noted between the HFB group and HF7 groups (P=0.037<0.05), with the HFB group predicted to have significantly more forms_biofilms bacteria than the HF7 group (**Figure 13**). BugBase attributes differences in the relative abundance of predicted forms_biofilms bacteria to the higher proportion of *Haemophilus* and *Neisseria (*phylum *Proteobacteria)* in the HFB group.

## Discussion

Stomatologists have paid increasing attention to the prevention and intervention of ECC, and the main preventive service is the topical application of fluoride varnish every 3 to 6 months. Our study group has demonstrated the effectiveness of fluoride in ECC prevention (Wang, 2019), but the microbial mechanism of dental caries prevention was not investigated. Microbiota are considered biomarkers for disease detection and management (Claesson et al., 2017; Kashyap et al., 2017). High-throughput techniques provide an effective way to study changes in the composition and structure of bacterial communities. In this study, we collected saliva samples at different times before and after fluoride treatment to study the changes in microbial populations, and the results helped us to have a comprehensive understanding of the impact of fluoride on the S-ECC oral microbiome, which may provide a theoretical basis for the clinical application of fluoride in S-ECC.

In this study, the alpha diversity index showed that the diversity and richness of bacterial communities in each time period before and after fluoride application were relatively stable and that species richness first increased, then decreased after fluoride use, and finally became comparable to prefluoride levels. As previous studies described, fluoride increased the diversity and richness of intestinal microflora in mice and silkworm 734 (Guannan et al., 2015; Fu et al., 2019; Liu et al., 2019) but decreased it in children with dental fluorosis, silkworm T6, and broiler chickens (Guannan et al., 2015; Luo et al., 2016; Wu, 2019). In conclusion, we speculate that the use of fluoride will affect oral microbial richness, that the post-use time of fluoride will also affect species richness and that the mechanism of the relationship between fluoride and the oral microbiota is related to the time after fluoride use.

From the community graph, the community structure of the groups was similar before and after fluoride use, indicating that the use of fluoride may not have a significant effect on the bacterial composition, and its main effect appears to be on the demineralization and remineralization processes in the oral cavity, which is similar to a previous study (Koopman et al., 2015). At the genus level, the most prevalent bacteria in the five groups were largely uniform but varied in relative abundance. The differences *in Bacteroides, Turicibacter* and *uncultured_bacterium_f_Enterobacteriaceae* among the five groups could be used as markers of microbial diversity changes following presumed fluoride use. The relative abundance of *Uncultured_bacterium_f_Enterobacteriaceae*, belonging to *Proteobacteria*, was higher in the HF14 group*. Proteobacteria* is a phylum generally associated with health, but *Turicibacte* belongs to *Firmicutes*, a phylum that consists of major cariogenic pathogens. This species was higher in the HFB group, which indicates that the use of local fluoride can reduce *Firmicutes*. Previous studies have reported comparative oral microbiome profiles between healthy individuals and patients with dental caries. Healthy individuals, especially children, had higher levels of *Proteobacteria* and *Bacteroidetes* and lower levels of *Firmicutes* in the oral microbiome (Bik et al., 2010; Chen et al., 2017). *Bacteroides* belongs to *Bacteroidetes*. A study (Wang et al., 2022) found that its change trend in the salivary microflora in children with caries was opposite that of *Firmicutes*, which was consistent with the results of this study. Hence, overall, active dental caries in children is likely to be associated with higher *Firmicutes* and lower *Proteobacteria* and *Bacteroidetes*. The findings of the present study provide new evidence that fluoride use could not only reduce cariogenic bacteria but also enrich the healthier microbiome in treated children.

Network analysis showed the potential correlation of oral microbiota. In this study, the analysis of different groups of microbiota clearly showed the different relationships among oral microbiota, and their relationships gradually changed from complex to simple to complex with time, which indicated that the use of fluoride led to ecological changes in oral microbiota. By analyzing the relationships between *Bacteroides* and other genera in the five groups, it can be concluded that the relationship between *Bacteroides* and other genera changed from complex to simple with the change in local fluoride usage time, and after fluoride usage, their relationships all became positively correlated. The phylum to which *Bacteroides* belongs is a health-related phylum, which indicates that with the change in local fluoride usage time, the microbial population in the oral cavity gradually tends to be healthy.

In this study, the functional analysis of salivary microbial communities before and after the use of local fluoride in children with high caries found that the starch and sucrose metabolism difference was statistically significant when carbohydrate metabolism was annotated to Class 3, and starch and sucrose metabolism was enriched in the HF1 groups, indicating that its changes helped to improve the oral microecology of children with high caries and move it towards a healthy oral microecology. The comparative analysis of the high-caries and healthy groups conducted by Liu (2021) found that carbohydrate metabolism (carbohydrate metabolism) was enriched in the healthy group. Wang et al. (2019) found that the functional difference between children with high caries and no caries was mainly reflected in carbohydrate metabolism, and our study found similar results. BugBase phenotype prediction follows pairwise comparisons between HF14 and HFB groups regarding forms_biofilms, with higher relative abundance of *Haemophilus* and *Neisseria* reflected in the HFB group than in the HF7 group, suggesting that *Haemophilus* and *Neisseria* may decrease at 7 days after local fluoride use. Johansson et al. reported that the oral microbiomes of individuals with high caries levels are dominated by *Streptococcus, Alloprevotella, Leptotrichia, Neisseria, Prevotella*, and *Porphyromonas*, while caries-free microbiomes are dominated by *Gemella* (Johansson et al., 2016). This is consistent with the results of this study, but there are also studies suggesting that (Liu, 2021) *Haemophilus* and *Neisseria* generally have high relative abundance in children in the healthy group. This study has shown that health-related *Bacteroides* increased after topical fluoride use at Day 14, but no statistically significant differences were found in colonies before and after topical fluoride use at Day 7, suggesting that different genera belonging to the same phylum level may show different changes and that the same genus also shows different changes in different individuals; thus, the oral microbial diversity is in a state of complex dynamic changes, and there are individual differences.

The main limitation of this study is the relatively small sample size, so an increase in the sample size in the future is encouraged. This study explored the effect of fluoride on the microbial diversity of oral saliva. However, there are many environmental conditions affecting microbial diversity in oral saliva, such as temperature, pH, salinity, redox potential, and the acquisition of oxygen or nutrients, so other environmental impact factors need to be strictly controlled to increase the accuracy and reliability of the results. In addition, the concentration of fluoride in saliva after fluoride varnish application is also a factor that affects the results. Eakle et al. (Eakle et al., 2004) found that the maximum fluoride levels in saliva with varnish remained above baseline levels for a longer duration, but further research is needed.

## Conclusion

The present study found that certain bacterial phylotypes of the saliva microbiome were significantly modulated by the local use of fluoride in S-ECC. It was observed that health-associated genera such as *Bacteroides* and *Uncultured_bacterium_f_Enterobacteriaceae* were enriched in the saliva microbiome following treatment with fluoride-containing material. The new findings highlight the need for a better understanding of the oral microbiome in the etiopathology of caries in children and evaluating the efficacy of dental treatments such as fluoride-containing materials, which will provide more favorable evidence for the use of fluoride in caries prevention and therapy in severe early childhood caries.

## Data availability statement

The datasets presented in this study can be found in online repositories. The names of the repository/repositories and accession number(s) can be found in the article/**Supplementary Material**.

## Ethics statement

The studies involving human participants were reviewed and approved by the Ethics Committee of the Stomatological Hospital of Chongqing Medical University(CQHS-REC-2018(LSNO.22). Written informed consent to participate in this study was provided by the participants’ legal guardian/next of kin.

## Author contributions

ZY, TC, and YL contributed to research design, data acquisition, data analysis and interpretation, and drafted and critically revised manuscript. DJ contributed to data interpretation and critically revised the manuscript. ZZ and JL contributed to conception and design and critically revised the manuscript. All authors agreed to be accountable for all aspects of this work. All authors contributed to the article and approved the submitted version.
